# Simulation der Letalität nach verschiedenen Ex-ante- und Ex-post-Triage-Verfahren bei Menschen mit Behinderungen und Vorerkrankungen

**DOI:** 10.1007/s00101-023-01302-3

**Published:** 2023-06-26

**Authors:** Sara Garber, Jens O. Brunner, Axel R. Heller, Georg Marckmann, Christina C. Bartenschlager

**Affiliations:** 1 Lehrstuhl für Health Care Operations/Health Information Management, Wirtschaftswissenschaftliche und Medizinische Fakultät, Universität Augsburg, Universitätsstr. 16, 86159 Augsburg, Deutschland; 2 Professor of Decision Science in Healthcare, Department of Technology, Management, and Economics, Technical University of Denmark, Lyngby, Dänemark; 3 Klinik für Anästhesiologie und Operative Intensivmedizin, Medizinische Fakultät, Universitätsklinikum Augsburg, Universität Augsburg, Stenglinstr. 2, 86156 Augsburg, Deutschland; 4 Institut für Ethik, Geschichte und Theorie der Medizin, Ludwig-Maximilians-Universität München, Lessingstr. 2, 80336 München, Deutschland; 5 Professur für Angewandte Datenwissenschaften im Gesundheitswesen, Nürnberg School of Health, Technische Hochschule Nürnberg Georg Simon Ohm, Klinikum Nürnberg, Nürnberg, Deutschland

**Keywords:** Ethik, Lebenswertgleichheit, Alter, Überlebenswahrscheinlichkeit, Scores, Ethics, Life value equality, Age, Probability of survival, Scores

## Abstract

Der stetige Anstieg an zu behandelnden Patienten während der COVID-19-Pandemie hat das Gesundheitssystem vor eine Vielzahl an Herausforderungen gestellt. Die Intensivstation ist einer der in diesem Zusammenhang besonders stark betroffenen Bereiche. Nur durch umfangreiche Infektionsschutzmaßnahmen sowie einen enormen logistischen Aufwand konnten in Deutschland selbst in Hochphasen der Pandemie die Behandlung aller Intensivpatienten ermöglicht und eine Triage auch in Regionen mit hohem Patientendruck bei gleichzeitig geringen Kapazitäten verhindert werden. Im Hinblick auf die Pandemievorsorge hat der Deutsche Bundestag ein Gesetz zur Triage verabschiedet, das eine Ex-post-Triage explizit untersagt. Bei einer Ex-post-Triage werden auch Patienten, die bereits auf der Intensivstation behandelt werden, in die Triage-Entscheidung einbezogen und Behandlungskapazitäten nach individueller Erfolgsaussicht verteilt. In der Literatur finden sich rechtliche, ethische und soziale Überlegungen zur Triage bei Pandemien, eine quantitative Bewertung im Hinblick auf verschiedene Patientengruppen auf der Intensivstation gibt es hingegen nicht. Der Fokus der Arbeit liegt auf dieser Forschungslücke, und es wird eine quantitative simulationsbasierte Evaluation von Ex-ante- und Ex-post-Triage-Politiken unter Berücksichtigung von Überlebenswahrscheinlichkeiten, Beeinträchtigungen und Vorerkrankungen durchgeführt. Die Ergebnisse zeigen, dass eine Anwendung von Ex-post-Triage, basierend auf Überlebenswahrscheinlichkeiten in allen Patientengruppen, zu einer Reduktion der Mortalität auf der Intensivstation führt. In dem Szenario, das der realen Situation wohl am nächsten kommt, wird eine Reduktion der Mortalität auf der Intensivstation um ca. 15 % schon bei einer einmaligen Anwendung der Ex-post-Triage erreicht. Dieser mortalitätsreduzierende Effekt ist umso größer, je mehr Patienten auf eine intensivmedizinische Behandlung warten.

**Zusatzmaterial online:**

Die Online-Version dieses Beitrags (10.1007/s00101-023-01302-3) enthält weitere Tabellen.

## Abstract (en)

The significant increase in patients during the COVID-19 pandemic presented the healthcare system with a variety of challenges. The intensive care unit is one of the areas particularly affected in this context. Only through extensive infection control measures as well as an enormous logistical effort was it possible to treat all patients requiring intensive care in Germany even during peak phases of the pandemic, and to prevent triage even in regions with high patient pressure and simultaneously low capacities. Regarding pandemic preparedness, the German Parliament passed a law on triage that explicitly prohibits ex post (tertiary) triage. In ex post triage, patients who are already being treated are included in the triage decision and treatment capacities are allocated according to the individual likelihood of success. Legal, ethical, and social considerations for triage in pandemics can be found in the literature, but there is no quantitative assessment with respect to different patient groups in the intensive care unit. This study addressed this gap and applied a simulation-based evaluation of ex ante (primary) and ex post triage policies in consideration of survival probabilities, impairments, and pre-existing conditions. The results show that application of ex post triage based on survival probabilities leads to a reduction in mortality in the intensive care unit for all patient groups. In the scenario close to a real-world situation, considering different impaired and prediseased patient groups, a reduction in mortality of approximately 15% was already achieved by applying ex post triage on the first day. This mortality-reducing effect of ex post triage is further enhanced as the number of patients requiring intensive care increases.

## Einleitung

Der stetige Anstieg an zu behandelnden Patienten^1^ während der COVID-19-Pandemie hat das Gesundheitssystem vor eine Vielzahl an Herausforderungen gestellt. Die Intensivstation ist einer der in diesem Zusammenhang besonders stark betroffenen Bereiche. Nur durch umfangreiche Infektionsschutzmaßnahmen sowie einen enormen logistischen Aufwand [1] konnten in Deutschland selbst in Hochphasen der Pandemie die Behandlung aller Intensivpatienten ermöglicht und eine Triage auch in Regionen mit hohem Patientendruck bei gleichzeitig geringen Kapazitäten verhindert werden [2]. Im Zusammenhang mit der Problematik nicht ausreichend zur Verfügung stehender Ressourcen wird oftmals von „Priorisierungsentscheidungen bei Ressourcenknappheit“ [3] gesprochen. In dieser Arbeit wird der Begriff der „Triage“ verwendet, der sich im Sprachgebrauch bis in den Bundestag und die Medien verfestigt hat.

Im Hinblick auf die Pandemievorsorge hat der Deutsche Bundestag ein Gesetz zur Triage verabschiedet, das eine Ex-ante-Triage nicht ausschließt, eine Ex-post-Triage hingegen explizit untersagt. Im Gesetzestext (§5c ISchG Abs. 2, Satz 4) heißt es dazu konkret: „Bereits zugeteilte überlebenswichtige intensivmedizinische Behandlungskapazitäten sind von der Zuteilungsentscheidung ausgenommen“. Bei einer Ex-post-Triage werden auch Patienten, die bereits auf der Intensivstation behandelt werden, in die Triage-Entscheidung einbezogen und Behandlungskapazitäten nach individueller Erfolgsaussicht verteilt [2, 3]. Dies kann dazu führen, dass die Behandlung eines Patienten auf der Intensivstation zugunsten der intensivmedizinischen Behandlung eines anderen Patienten nicht fortgesetzt wird. Ein Ziel des neuen Gesetzes ist, eine Benachteiligung von Patienten mit Beeinträchtigungen und Vorerkrankungen zu verhindern. Zudem darf lediglich die aktuelle und kurzfristige Überlebenswahrscheinlichkeit bei der Zuteilung von Intensivkapazitäten berücksichtigt werden [4].

In der Literatur finden sich rechtliche, ethische und soziale Überlegungen zur Triage bei Pandemien [3, 5–9], eine quantitative Bewertung im Hinblick auf verschiedene Patientengruppen auf der Intensivstation gibt es hingegen nicht. Der Fokus der Arbeit liegt auf dieser Forschungslücke, und es wird eine quantitative simulationsbasierte Evaluation der Auswirkungen verschiedener Steuerungspolitiken auf der Intensivstation durchgeführt. Mithilfe eines Simulationsmodells werden die Effekte verschiedener Ex-ante- und Ex-post-Triage-Politiken auf die Mortalität auf der Intensivstation evaluiert. Der Fokus liegt hierbei insbesondere auf den Auswirkungen für unterschiedliche Patientengruppen, d. h. Patienten ohne Beeinträchtigungen und Vorerkrankungen sowie Patienten mit Beeinträchtigungen und Vorerkrankungen. Die Arbeit basiert auf Daten aus der Literatur und bietet Unterstützung für Entscheidungsträger in der Planung und Steuerung von Intensivkapazitäten in Hochlastsituationen, in welchen eine lebensnotwendige medizinische Versorgung nicht mehr allen Patienten ermöglicht werden kann.

## Methoden

**Figure 1:**
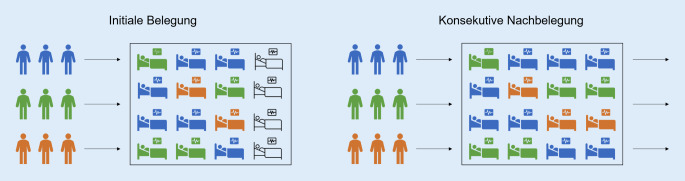

In der Studie wurde eine Intensivstation mit COVID-19-Patienten simuliert. Dabei wurde zwischen Patienten mit bzw. ohne zusätzliche Komorbidität oder Behinderung unterschieden. Die Anzahl der zu behandelnden Patienten überstieg die Anzahl an betreibbaren Intensivbehandlungsplätzen, d. h., es musste entschieden werden, welche Patienten eine Intensivkapazität erhalten konnten und bei welchen Patienten keine Behandlung (mehr) erfolgen konnte (Abb. 1). Dabei war ein ICU-Bett definiert als personell und apparativ voll ausgestattete ICU Behandlungskapazität die Organunterstützung und ggf. -Ersatz für jeweils einen Patienten leisten kann.

Die Entscheidung, welche Patienten (weiter)behandelt werden konnten, wurde je nach Steuerungspolitik zufällig oder basierend auf Überlebenswahrscheinlichkeiten getroffen. Der Fokus lag hierbei auf den Auswirkungen für unterschiedlichen Patientengruppen auf der Intensivstation. Im folgenden Kapitel werden der zugrunde liegende Datensatz, die untersuchten Triage-Politiken, d. h. Steuerungspolitiken, sowie das Simulationsmodell und dessen Evaluation beschrieben.

### Datensatz

Diese Studie basierte auf Daten aus der Literatur. Bei Patienten ohne Beeinträchtigungen und Vorerkrankungen wurden zur Generierung der Sterbewahrscheinlichkeiten die (normierten) Werte des Robert Koch-Instituts (RKI) verwendet [10]. Die Sterbewahrscheinlichkeit entsprach dabei dem Komplement zur Überlebenswahrscheinlichkeit. Es wurde eine Dreiecksverteilung angenommen, da hier die vom RKI [10] identifizierten Maße aus einer Anwendersicht intuitiv nachvollziehbar implementiert werden konnten^2^:

$$X\ \Updelta (0{,}04;0{,}41;0{,}045)$$

mit^3^:

$$E\left(x\right)=0{,}165$$

Die Dreiecksverteilung, deren Name ihre Visualisierung verbalisiert, wurde dabei über den minimalen, maximalen und wahrscheinlichsten Wert (Modus) definiert. Anschließend wurden (adjustierte) literaturbasierte Verhältnismaße für das (approx.) relative Sterberisiko [11–15], d. h. Odds Ratio und Hazard Ratio, auf die Verteilung bzw. deren Erwartungswert angewendet. Daraus ergaben sich die Dreiecksverteilungen zur Generierung der Sterbewahrscheinlichkeiten von Patienten mit Beeinträchtigungen und Vorerkrankungen. In der Simulation wurden 2 Beeinträchtigungen, d. h. Trisomie 21 und amyotrophe Lateralsklerose, sowie 3 Vorerkrankungen, d. h. Herz-Kreislauf-Erkrankung, Hypertonie und Diabetes mellitus (Typ 2), betrachtet. Bei der Betrachtung von Beeinträchtigungen wurde sowohl die amyotrophe Lateralsklerose gewählt, da diese Erkrankung eine zentrale Rolle in den Argumentationen gegen eine Ex-post-Triage innehatte, als auch die Trisomie 21, da für diese Beeinträchtigung ebenfalls Verhältnismaße in Bezug auf COVID-19-Risikofaktoren in der Literatur zu finden sind. Als Odds Ratio bzw. Hazard Ratio wurden folgende Werte verwendet: Risiko für eine Behandlung auf der ITS (Trisomie 21), 30-Tage-Sterberisiko (amyotrophe Lateralsklerose), Krankenhaussterblichkeit (Herz-Kreislauf-Erkrankung), relatives Sterberisiko (Hypertonie) und Risiko für Tod im Krankenhaus durch COVID-19 (Diabetes mellitus, Typ 2). Zur Vereinfachung werden diese Werte im Folgenden als relatives Sterberisiko bezeichnet. Das Verhältnismaß für das relative Sterberisiko ist unter den betrachteten Beeinträchtigungen und Vorerkrankungen bei einer Herz-Kreislauf-Erkrankung mit 4,85 am höchsten, während dieses bei Diabetes mellitus (Typ 2) mit 2,03 am niedrigsten ist. Eine Übersicht aller verwendeten, literaturbasierten Verhältnismaße für das relative Sterberisiko ist in Tab. 1 dargestellt.

**Table 1:** 

Schwerpunkt	Paper	Verhältnismaß	Verwendete Werte
Trisomie 21	Bergman et al. (2021) [11]	Odds Ratio	$4{,}52;KI(3{,}13;7{,}36)$^a^
Herz-Kreislauf-Erkrankung	Li et al. (2020) [12]	Odds Ratio	$4{,}85;KI(3{,}07;7{,}70)$
Hypertonie	Gao et al. (2020) [13]	Hazard Ratio	$2{,}12;KI(1{,}17;3{,}82)$
Diabetes mellitus (Typ 2)	Barron et al. (2020) [14]	Odds Ratio	$2{,}03;KI(1{,}97;2{,}09)$
Amyotrophe Lateralsklerose	Galea et al. (2021) [15]	Odds Ratio	$3{,}0;KI(1{,}9;4{,}9)$

^a^Reduktion des *KI* um 40*%*

**Figure 2:**
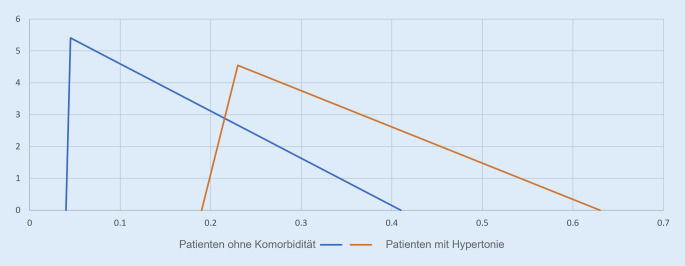

Es ist zu beachten, dass die Parameter der Dreiecksverteilung, d. h. Minimum, Maximum und Modus, zur Generierung der Sterbewahrscheinlichkeit für Patienten mit Beeinträchtigungen und Vorerkrankungen so festgelegt wurden, dass eine Anwendung der Verhältnismaße auf den Erwartungswert möglich war. Gleichzeitig wurde sichergestellt, dass beeinträchtigte und vorerkrankte Patienten eine niedrigere Überlebenswahrscheinlichkeit als Patienten ohne Beeinträchtigungen und Vorerkrankungen haben konnten. In Abb. 2 sind beispielhaft die Dichtefunktionen der Sterbewahrscheinlichkeiten^4^ von Patienten ohne Beeinträchtigung oder Vorerkrankung und Patienten mit Hypertonie dargestellt.

Bei Anwendung des Verhältnismaßes ergab sich für Patienten mit Hypertonie folgende Dreiecksverteilung:

$$X\ \Updelta (0{,}19;0{,}63;0{,}23)$$

mit

$$E\left(X\right)=0{,}35$$

Der Anteil des sich überschneidenden Flächeninhalts der beiden Dichtefunktionen betrug in diesem Fall somit 31,35 %.

### Triage-Politiken

Für den Vergleich der Auswirkungen verschiedener Steuerungspolitiken auf die Mortalität auf der Intensivstation wurden verschiedene Kombinationen aus Ex-ante- und Ex-post-Triage^5^ simuliert. Als entscheidendes Kriterium für die Triage-Entscheidung wurde die Sterbewahrscheinlichkeit (Gegenwahrscheinlichkeit zur Überlebenswahrscheinlichkeit) verwendet. Die Berücksichtigung der Überlebenswahrscheinlichkeit, die auf Basis von medizinischen Scores berechnet werden kann und laut dem aktuellen Gesetz als einziges Kriterium herangezogen werden darf, liefert im Vergleich zu anderen Kriterien wie beispielsweise dem Alter ein superiores Ergebnis [2]. In der Simulation wurden Verfahren der initialen Belegung, d. h. initiale zufällige Belegung und initiale Ex-ante-Triage auf Basis von Überlebenswahrscheinlichkeiten, mit Verfahren der konsekutiven Nachbelegung, d. h. zufällige konsekutive Nachbelegung und konsekutive Ex-post-Triage, basierend auf Überlebenswahrscheinlichkeiten, kombiniert. Die einzelnen Kombinationen in den jeweiligen Steuerungspolitiken können Tab. 2 entnommen werden.

**Table 2:** 

Politik	Beschreibung
0	Initiale zufällige Belegung, zufällige konsekutive Nachbelegung
1	Initiale zufällige Belegung, konsekutive Ex-post-Triage auf Basis von Überlebenswahrscheinlichkeiten
2	Initiale Ex-ante-Triage auf Basis von Überlebenswahrscheinlichkeiten, zufällige konsekutive Nachbelegung
3	Initiale Ex-ante-Triage auf Basis von Überlebenswahrscheinlichkeiten, konsekutive Ex-post-Triage auf Basis von Überlebenswahrscheinlichkeiten
4	90 % initiale zufällige Belegung/10 % initiale Ex-ante-Triage auf Basis von Überlebenswahrscheinlichkeiten, zufällige konsekutive Nachbelegung (entsprechend aktueller Gesetzeslage)
5	90 % initiale zufällige Belegung/10 % initiale Ex-ante-Triage auf Basis von Überlebens-wahrscheinlichkeiten, konsekutive Ex-post-Triage auf Basis von Überlebenswahrscheinlichkeiten

Bei Politik 0 handelte es sich um die Darstellung eines Szenarios, in dem weder eine Ex-ante- noch eine Ex-post-Triage auf Basis von Überlebenswahrscheinlichkeiten durchgeführt wurde. Die Politik diente somit als Benchmark für alle weiteren Steuerungspolitiken. Bei den Politiken 1 und 2 wurde jeweils nur eine Form der Triage auf Basis von Überlebenswahrscheinlichkeiten durchgeführt, wobei Politik 2 eine untere Grenze für die Mortalität auf der Intensivstation lieferte, da eine initiale Ex-ante-Triage auf Basis von Überlebenswahrscheinlichkeiten zur Belegung der kompletten Intensivstation in der Praxis wohl nie durchgeführt wird. Nichtsdestotrotz vermochte Politik 2 den maximalen Effekt darzustellen, der durch die Anwendung einer initialen Ex-ante-Triage auf Basis von Überlebenswahrscheinlichkeiten erzielt werden konnte. Eine Kombination der Ex-ante- und Ex-post-Triage auf Basis von Überlebenswahrscheinlichkeiten wurde bei Politik 3 abgebildet. Zusätzlich wurden zur realitätsnahen Darstellung 2 weitere Politiken, 4 und 5, eingeführt. Hier wurde von einer bereits zu 10 % gefüllten Intensivstation (zufällige Belegung) sowie einer konsekutiven Ex-ante-Triage auf Basis von Überlebenswahrscheinlichkeiten zur Belegung der restlichen 10 % der verfügbaren Betten ausgegangen. Darauf basierend erfolgte entweder eine zufällige konsekutive Nachbelegung (Politik 4), welche einem Vorgehen nach dem aktuellen Gesetz wohl am nächsten kommt, oder eine konsekutive Ex-post-Triage auf Basis von Überlebenswahrscheinlichkeiten (Politik 5).

### Simulationsmodell und Evaluation

Für die Simulationsstudie in der Statistiksoftware R wurden für die 6 betrachteten Steuerungspolitiken jeweils 6 verschiedene Simulationen durchgeführt. Diese unterscheiden sich jeweils in der Betrachtung verschiedener Patientengruppen bzw. deren Kombination. So wurden jeweils eine Simulation für jede der 5 betrachteten Komorbiditäten unter der Annahme, dass 30 % der Patienten nicht beeinträchtigt oder vorerkrankt waren und 70 % der Patienten an der berücksichtigten Beeinträchtigung oder Vorerkrankung litten [16], sowie eine Realsimulation durchgeführt. In der Realsimulation konnten innerhalb der Patientengruppe mit Komorbiditäten alle betrachteten Beeinträchtigungen und Vorerkrankungen auftreten. Dieses Vorgehen ermöglichte sowohl eine individuelle Beurteilung der Auswirkungen einer bestimmten Beeinträchtigung oder Vorerkrankung als auch die Evaluation eines realitätsnahen Szenarios, in welchem unterschiedliche Patientengruppen auf der Intensivstation behandelt wurden.

Im Folgenden wird der Aufbau der Realsimulation dargelegt, da in dieser alle Komorbiditäten berücksichtigt werden. Der Ablauf der anderen Simulationen ist größtenteils äquivalent, allerdings erfolgt keine Unterscheidung innerhalb der Patientengruppe mit Beeinträchtigungen und Vorerkrankungen. Zu Beginn der Realsimulation wurden die Anzahl an Simulationsdurchläufen, $R=10.000$, Steuerungspolitiken, $P=6$, betrachteten Zeitpunkten, $T=3$ 6, der Bedarf zum Zeitpunkt der Initialisierung, $d=70{,}$ sowie der betrachteten Länge der Warteschlange, $w=10$, verfügbaren Intensivbetten, $B=60$, sowie der Anteil an Patienten mit Beeinträchtigung oder Vorerkrankung, $s=0{,}7$, vorab festgelegt. Die Betrachtung von $B=60$ Betten basierte auf den voll betreibbaren Intensivkapazitäten am Universitätsklinikum Augsburg in einer COVID-19-Hochlastsituation. In jedem Simulationsdurchlauf erfolgte nach der Initialisierung zunächst die Generierung der Patienten, aus welchen die Initialbelegung der Intensivstation ermittelt wurde. Im Fall einer initialen zufälligen Belegung wurden zufällig 60 Patienten generiert, im Fall einer initialen Ex-ante-Triage, basierend auf Überlebenswahrscheinlichkeiten, wurde der Bedarf zum Zeitpunkt der Initialisierung im Sinne derer Patienten, die eine intensivmedizinische Behandlung nachfragten, generiert^7^. Für jeden Patienten $i\in I$ erfolgte die Zuordnung einer Sterbewahrscheinlichkeit, basierend auf einer Dreiecksverteilung, abhängig davon, ob bzw. welche Beeinträchtigung oder Vorerkrankung *k* vorlag:

$$p_{i}\ \Updelta \left({a}_{i}^{k}{,}{b}_{i}^{k}{,}{c}_{i}^{k}\right)\ \forall i\in I$$

Es wurde somit eine Abhängigkeit modelliert, ob der Patient eine bzw. welche Komorbidität er aufwies^8^. Zusätzlich wurde *x*_*i*_ eingeführt, um anzugeben, ob ein Patient beeinträchtigt oder vorerkrankt war:

$$x_{i}=\begin{cases} 1 & \text{wenn Patient } i \text{ beeintr{\"a}chtigt}\\ & \text{oder vorerkrankt ist}\\ 0 & \text{sonst} \end{cases}$$

**Figure 3:**
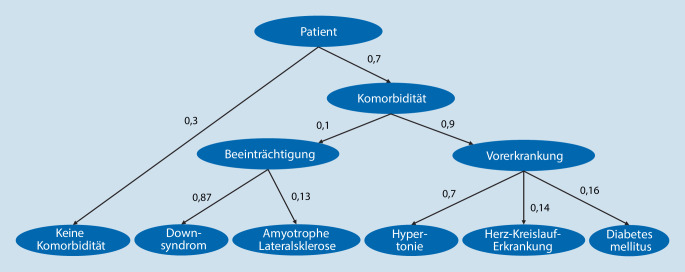

Im Fall eines Patienten mit einer Komorbidität wurden, basierend auf den (adjustierten) Prävalenzen, die Beeinträchtigungen und Vorerkrankungen zugeordnet ([17–23]; Abb. 3). Es wurde angenommen, dass ein solcher Patient mit einer Komorbidität mit einer Wahrscheinlichkeit von 10 % an einer Beeinträchtigung und mit einer Wahrscheinlichkeit von 90 % an einer Vorerkrankung litt. Dadurch wurde sichergestellt, dass alle betrachteten Komorbiditäten in der Realsimulation berücksichtigt wurden.

Basierend auf der zugeordneten Wahrscheinlichkeit wurde sodann über eine gleichverteilte Zufallszahl im Intervall $[0{,}1]$ eine binäre Variable *y*_*i*_ zugeordnet, welche angab, ob ein Patient im Fall einer intensivmedizinischen Behandlung verstarb:

$$y_{i}=\begin{cases} 1 & \text{wenn Patient }i\text{ im Fall einer}\\ & \text{Behandlung verstirbt}\\ 0 & \text{sonst} \end{cases}$$

Die Zuordnung dieser Variable erfolgte auf Basis der simulierten Sterbewahrscheinlichkeit.

Im Fall der initialen Ex-ante-Triage auf Basis von Überlebenswahrscheinlichkeiten wurden nun die Patienten mit den höchsten Überlebenschancen bzw. den niedrigsten Sterbewahrscheinlichkeiten für die intensivmedizinische Behandlung ausgewählt. Nach der initialen Belegung ($t=0$) wurde in jedem der betrachteten konsekutiven Zeitpunkte $t\in T=\{1{,}2{,}3\}$ eine Warteschlange generiert und entweder eine zufällige Nachbelegung oder eine Ex-post-Triage, basierend auf Überlebenswahrscheinlichkeiten, durchgeführt. Bei einer zufälligen Nachbelegung wurden zufällig jeweils 6 Patienten (10 % der Intensivkapazität) auf der Intensivstation und in der Warteschlange ausgewählt, die Intensivkapazität freigeben bzw. erhalten. Bei einer konsekutiven Ex-post-Triage, basierend auf Überlebenswahrscheinlichkeiten, erfolgte die Zuteilung der Intensivkapazitäten nach den generierten Sterbewahrscheinlichkeiten. Für jeden der betrachteten Zeitpunkte erfolgte eine Berechnung der prospektiven Mortalität auf der Intensivstation für die im jeweiligen Zeitpunkt behandelte Patientenkohorte:

$$m_{t}=\frac{1}{B}\cdot \sum _{i\in I}y_{i}\ \forall t\in T$$

Die Berechnung der Mortalität innerhalb der einzelnen Patientengruppen, d. h. Patienten ohne Beeinträchtigungen und Vorerkrankungen (${m}_{t}^{n}$) sowie Patienten mit Beeinträchtigungen und Vorerkrankungen (${m}_{t}^{v}$), erfolgte äquivalent. Zusätzlich wurde die Anzahl der behandelten, entlassenen und aufgenommenen Patienten in jeder Patientengruppe zu jedem Zeitpunkt berechnet. Im Anschluss wurden die Mittelwerte sowie die Standardabweichungen aller betrachteten Kennzahlen berechnet. Zusätzlich wurden zur Validierung der Ergebnisse ANOVA (Analysis of Variance) und Post-hoc-Tests durchgeführt.

Um den Einfluss der Anzahl an zu behandelten Patienten darzustellen und somit die Sensitivität zu evaluieren, wurden unterschiedliche Kombinationen des Bedarfs zum Zeitpunkt der Initialisierung, $d\in D=\{70{,}90\}$, und der Warteschlangenlänge, $w\in W=\{10{,}20{,}30{,}60\}$, betrachtet. Um die Auswirkungen potenzieller Fehleinschätzungen zu evaluieren, wurde außerdem mithilfe des Parameters $e\in E=\{0{,}9;1;1{,}1\}$ angegeben, ob die Einschätzung der Sterbewahrscheinlichkeit der tatsächlichen Wahrscheinlichkeit entsprach ($e=1$) oder ob diese von den behandelten Ärzten um 10 % unter- bzw. überschätzt wurde. Daraus ergab sich die Wahrscheinlichkeit, welche in der Triage-Entscheidung berücksichtigt wurde:

$$p\ _{i}=e\cdot p_{i}\ \forall e\in E{,}i\in I$$

**Figure 4:**
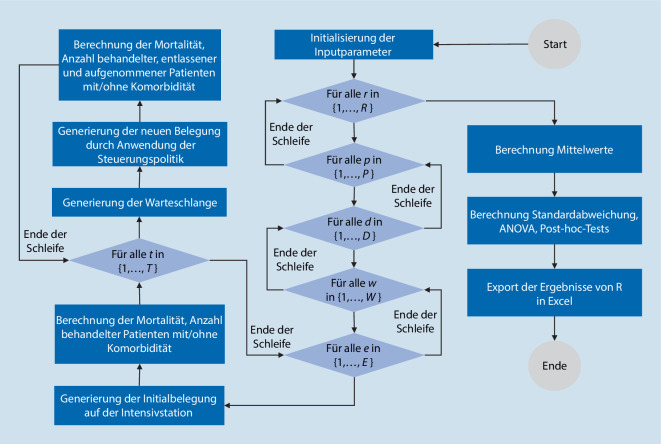

Eine Übersicht der Implementierung in R ist in Abb. 4 als Flussdiagramm dargestellt.

## Ergebnisse

**Figure 5:**
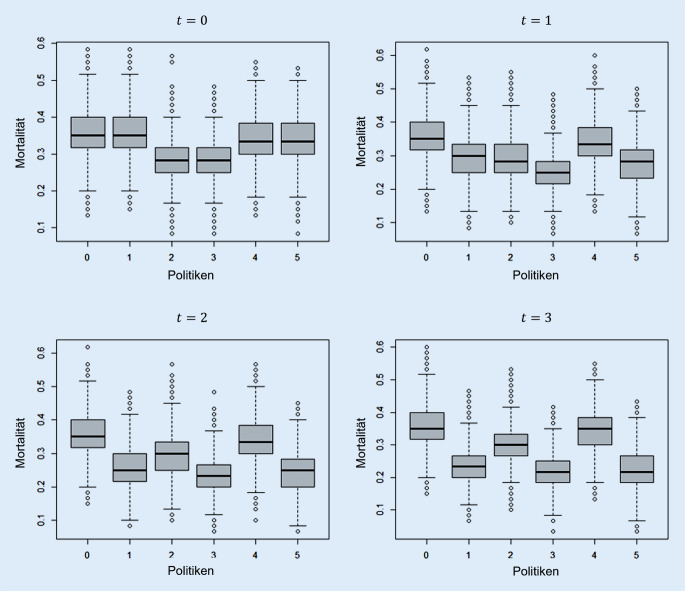

In dieser Simulationsstudie wird die Mortalität auf der Intensivstation bei der Anwendung unterschiedlicher Steuerungspolitiken in Hochlastsituationen berechnet. Im Folgenden werden die Ergebnisse für die Realsimulation bei einem Bedarf zum Zeitpunkt der Initialisierung von 70 Patienten (initiale Warteschlange), einer Warteschlangenlänge von 10 (jeweils in den Zeitpunkten 1, 2 und 3) und einer korrekten Einschätzung der Mortalität, d. h. $e=1$, dargestellt. Die gesamte durchschnittliche Mortalität auf der Intensivstation für jeden betrachteten Zeitpunkt und jede Steuerungspolitik ist in Abb. 5 dargestellt.

Zum Zeitpunkt der Initialisierung gibt es keine Unterschiede in der Mortalität bei Anwendung der Politiken 0 und 1, 2 und 3 sowie 4 und 5, da die Initialbelegung in diesen Politiken äquivalent erfolgt. Bei Betrachtung der konsekutiven Zeitpunkte zeigt sich, dass die Anwendung einer konsekutiven Ex-post-Triage, basierend auf Überlebenswahrscheinlichkeiten, zu einer Reduktion der (gesamten) Mortalität auf der Intensivstation führt. Bereits am ersten Tag ($t=1$) wird in Politik 5 eine Verringerung der Gesamtmortalität von 33,81 % auf 27,87 % erzielt. Hierbei handelt es sich um eine Reduktion der Mortalität um ca. 18 %. Eine Übersicht der Mortalität in den einzelnen Patientengruppen je Steuerungspolitik für den Zeitpunkt $t=1$ (erster Ex-post-Triage-Zeitpunkt) ist in Tab. 3 dargestellt. Die Ergebnisse zur Mortalität in den anderen Zeitpunkten, deren Standardabweichungen sowie die Anzahl an behandelten Patienten je Gruppe sind im elektronischen Anhang zu finden (Zusatzmaterial online: Tabellen S1 und S2).

**Table 3:** 

Politik	0 (in %)	1 (in %)	2 (in %)	3 (in %)	4 (in %)	5 (in %)
Alle Patienten	35,4	29,2	29,0	25,3	34,1	27,9
Patienten ohne Beeinträchtigung/Vorerkrankung	15,9	15,7	16,1	16,0	15,7	15,6
Patienten mit Beeinträchtigung/Vorerkrankung	43,8	36,4	35,8	31,5	42,9	35,3

Bei der Evaluation der untersuchten Variationen und Kennzahlen zeigen sich folgende Ergebnisse: Erstens ist bei den Variationen des Bedarfs zum Zeitpunkt der Initialisierung (initial wartende Patienten auf Intensivbehandlung zum Zeitpunkt 0) und der Warteschlangenlänge (wartende Patienten auf Intensivbehandlung zu den Zeitpunkten 1,2 und 3) zu beobachten, dass eine Triage bei einer zunehmenden Anzahl an zu behandelnden Patienten zu einer stärkeren Reduktion der Mortalität führt. Zweitens hat eine Variation des Parameters *e*, welcher angibt, ob die Sterbewahrscheinlichkeit des Patienten von den behandelten Ärzten richtig eingeschätzt oder um 10 % unter- bzw. überschätzt wurde, keinen signifikanten Einfluss auf die Mortalität auf der Intensivstation. Dies liegt darin begründet, dass die Sterbewahrscheinlichkeit in diesen Szenarien durchgehend unter-/über- bzw. richtig eingeschätzt wird. Drittens zeigt sich bei den Einzelsimulationen, in denen eine konkrete Beeinträchtigung oder Vorerkrankung berücksichtigt wird, dass die mortalitätsreduzierenden Effekte der Triage größer werden, je größer die Differenz zwischen den Erwartungswerten der Patientengruppen ist. Viertens zeigen die Ergebnisse der ANOVA, welche zur zusätzlichen Validierung der Ergebnisse durchgeführt wurden, statistisch signifikante Unterschiede. Die Post-hoc-Tests verdeutlichen, dass sich die Mittelwerte bei allen paarweisen Vergleichen signifikant unterscheiden. Eine Ausnahme stellt, wie zu erwarten, die initiale Belegung dar, da die Mittelwerte zwischen den Politiken, in denen die gleiche initiale Triage durchgeführt wird, nicht signifikant verschieden sind.

## Diskussion

Die Ergebnisse zeigen, dass die Durchführung einer konsekutiven Ex-post-Triage, basierend auf Überlebenswahrscheinlichkeiten, sowohl zu einer Reduktion der Gesamtmortalität auf der Intensivstation als auch der Mortalität innerhalb der einzelnen Patientengruppen auf der Intensivstation führt. Eine Ex-ante-Triage, basierend auf Überlebenswahrscheinlichkeiten, führt zwar zum Zeitpunkt der Initialisierung zu einer geringeren Mortalität auf der Intensivstation, allerdings steigt diese im Zeitverlauf wieder an, wenn lediglich eine zufällige Nachbelegung durchgeführt wird. Eine Kombination aus Ex-ante- und Ex-post-Triage, basierend auf Überlebenswahrscheinlichkeiten, liefert die besten Ergebnisse, allerdings ist hier zu beachten, dass eine reine Ex-ante-Triage in der Praxis wohl nie durchgeführt werden kann, da die Belegung einer Intensivstation dynamisch erfolgt. Selbst eine Ex-ante-Triage bei wenigen freien Betten ist wohl nur in seltenen Fällen möglich, da die Patienten im Zeitverlauf eintreffen und sequenziell über sie entschieden wird. Auch bei der Betrachtung der einzelnen Patientengruppen lassen sich Vorteile in Bezug auf die Mortalität auf der Intensivstation erkennen. Allerdings ist zu beachten, dass Patienten mit Beeinträchtigungen und Vorerkrankungen bei der Durchführung einer konsekutiven Ex-post-Triage, basierend auf Überlebenswahrscheinlichkeiten, überproportional entlassen bzw. nicht aufgenommen werden. Dies liegt darin begründet, dass der Erwartungswert der Sterbewahrscheinlichkeit bei dieser Patientengruppe größer ist als bei Patienten ohne Beeinträchtigungen und Vorerkrankungen. Auch wenn eine Ex-post-Triage, basierend auf Überlebenswahrscheinlichkeit, zu einer Reduktion der Mortalität auf der Intensivstation in allen betrachteten Patientengruppen führt, werden tendenziell mehr Patienten mit Beeinträchtigungen und Vorerkrankungen nicht (mehr) behandelt. Allerdings ist zu beachten, dass laut Literaturangaben 70 % der Patienten auf der Intensivstation beeinträchtigt oder vorerkrankt sind [16], weshalb in dieser Gruppe im Vergleich zu Patienten ohne Beeinträchtigungen und Vorerkrankungen stets eine größere Anzahl an Patienten nicht (weiter)behandelt werden kann. Außerdem wird in der Simulation deutlich, dass sich die mortalitätsreduzierenden Effekte der Triage verstärken, wenn die Anzahl an zu behandelnden Patienten, d. h. der Bedarf zum Zeitpunkt der Initialisierung und die Warteschlangenlänge, steigt. Schließlich wird in dieser Arbeit die Senkung der Mortalität als wesentliches Ziel erachtet, auch wenn es Akteure gibt, die eine höhere Mortalität zugunsten eines Losverfahrens oder anderer Verteilungsverfahren ethisch begründet in Kauf nehmen.

Die Studie unterliegt einigen Einschränkungen. Erstens wird die Annahme getroffen, dass alle Patienten, welchen keine sofortige Behandlung ermöglicht werden kann, versterben. Dadurch liegt der Fokus ausschließlich auf der Mortalität auf der Intensivstation. Zweitens wird in unserem Modell zur Abbildung der Hochlastsituation ein natürlicher Patientenfluss, d. h. die Möglichkeit einer (Ab‑)Verlegung von Patienten, bedingt durch eine Verbesserung des Zustands oder aufgrund von Todesfällen, ausgeschlossen. Drittens verändert sich die Überlebenswahrscheinlichkeit eines Patienten während seiner Behandlung auf der Intensivstation nicht. In der Praxis kann sich diese, ggf. angezeigt durch medizinische Scores, während des Behandlungsverlaufs jedoch verändern und somit würde eine auf diesen Scores basierende Steuerung zu anderen Ergebnissen kommen. Zunächst werden Patienten auf der ITS unter der Annahme behandelt, dass eine Intensivtherapie sie erfolgreich ins Leben zurückführt. Bei vielen Patienten verschiebt sich die Überlebenswahrscheinlichkeit jedoch erheblich – sowohl in die eine als auch in die andere Richtung. Eine Priorisierungsentscheidung in einem konsekutiven Zeitpunkt ist außerordentlich schwierig, es sei denn, eine medizinische Indikation zur Fortführung der Therapie ist nicht mehr gegeben, oder der Patientenwille richtet sich gegen die Fortführung einer ITS-Therapie. Zudem ergaben sich während der COVID-19-Pandemie deutlich längere Behandlungszeiten. Viertens werden die Parameter der Dreiecksverteilungen in der Simulation so gewählt, dass eine systematische Benachteiligung beeinträchtigter und vorerkrankter Patienten ausgeschlossen wird. Diese Annahme muss aus einer praktischen Sicht evaluiert werden. Fünftens entspricht die Anwendung einer reinen Ex-ante-Triage bei der Intensivstationsbelegung keinem realistischen Szenario, da eine Intensivstationsbelegung im Betrieb organisch wächst. Hierbei erfolgt die Verteilung frei werdender Kapazitäten nach Intensivbehandlungsnotwendigkeit der Patienten und nicht nach Erfolgsaussicht. Nur in Ausnahmefällen treffen mehrere Patienten gleichzeitig auf der Intensivstation ein und konkurrieren im Sinne einer Ex-ante-Triage um leere Intensivbehandlungsplätze. Die Vorstellung vieler bereitstehender leerer Intensivbehandlungsplätze und eines Ansatzpunkts für eine Ex-ante-Triage ist daher unwahrscheinlich. Sechstens wird in der Studie, basierend auf Charakteristika von Patienten in deutschen Krankenhäusern, die Annahme getroffen, dass 30 % der Patienten auf der Intensivstation nicht beeinträchtigt oder vorerkrankt sind. Zudem werden die (adjustierten) Prävalenzen verschiedener Beeinträchtigungen und Vorerkrankungen in Deutschland verwendet. Folglich ist eine Übertragung der Ergebnisse auf andere Regionen (unterschiedliche Patientengruppen, abweichende Prävalenzen) nur bedingt möglich.

## Schlussfolgerung

Diese Studie bietet eine simulationsbasierte Evaluation verschiedener Kombinationen aus Ex-ante- und Ex-post-Triage-Politiken auf der Intensivstation unter Berücksichtigung von Überlebenswahrscheinlichkeiten sowie Beeinträchtigungen und Vorerkrankungen. Die Ergebnisse zeigen, dass die Durchführung einer konsekutiven Ex-post-Triage auf Basis von Überlebenswahrscheinlichkeiten zu einer Reduktion der Mortalität auf der Intensivstation bei allen betrachteten Patientengruppen führt. Der Ausschluss der Ex-post-Triage ist daher kritisch auch im Lichte des aktuellen Gesetzes zu diskutieren.

Das Ziel dieser Arbeit ist, simulationsbasiert die Auswirkungen der Ex-ante- und Ex-post-Triage zu untersuchen. In der zukünftigen Forschung vermag der Ansatz der datengetriebenen Entscheidungsunterstützung bei der Steuerung von Intensivkapazitäten in Hochlastsituationen weiterverfolgt werden, wobei eine adäquate Datengrundlage von zentraler Bedeutung ist. Zusätzlich ist es essenziell, die Ergebnisse nicht isoliert zu betrachten, sondern interdisziplinär aus einer medizinisch-praktischen, ethischen und juristischen Perspektive zu diskutieren.

## Supplementary Information





## Footnotes

1 Aus Gründen der Lesbarkeit wurde das generische Maskulinum gewählt. Selbstverständlich beziehen sich alle Angaben auf Angehörige aller Geschlechter.

2 Minimum (0,04) Maximum (0,41) und Modus (0,045) der Sterbewahrscheinlichkeit.

3 Erwartungswert.

4 Eine Dichtefunktion beschreibt die Verteilung von Merkmalsausprägungen einer stetigen Variable. Die Fläche unterhalb der gegebenen Dichtefunktion beschreibt die Wahrscheinlichkeit, dass die Sterbewahrscheinlichkeit höchstens einen bestimmten Wert annimmt. So ist etwa die Wahrscheinlichkeit, dass die Sterbewahrscheinlichkeit von Patienten mit Hypertonie zwischen 0,19 und 0,63 liegt, 100 %.

5 Es erfolgt keine Evaluation einer sequenziellen Ex-ante-Triage, bei der (konsekutiven) Ex-post-Triage werden hingegen mehrere Zeitpunkte betrachtet.

6 Die initiale Belegung der Intensivstation erfolgt vor der Betrachtung der einzelnen Zeitpunkte und wird im Folgenden mit $t=0$ bezeichnet. Bei der Initiierung wird keine Ex-post-Triage durchgeführt.

7 Bei einer Kombination der beiden initialen Belegungsformen in den Politiken 4 und 5 wurde für die über eine Ex-ante-Triage, basierend auf Überlebenswahrscheinlichkeiten, zu belegenden Betten ein Bedarf von 12 Patienten angenommen.

8 Zur Validierung der Ergebnisse wurden die Sterbewahrscheinlichkeit für jede Patientengruppe 1000-mal simuliert und die Erwartungswerte berechnet. Diese entsprachen den errechneten Erwartungswerten der Dreiecksverteilungen bei Anwendung der Verhältnismaße.
