# Supplementary material for: Simulation of mortality after different ex-ante and ex-post-triage methods in people with disabilities and comorbidities
Source: Anaesthesiologie. 2023 Jun 26;72(8):555–64. [Article in German] doi: 10.1007/s00101-023-01302-3 (PMC10400691; doi:10.1007/s00101-023-01302-3)
Supplement: Supplementary file 1 [file 101_2023_1302_MOESM1_ESM.pdf]

Zusatzmaterial zum Beitrag **„Eine Simulationsstudie zur Evaluation von Ex-ante- und Ex-post-Triage-Politiken unter Berücksichtigung von Überlebenswahrscheinlichkeiten, Beeinträchtigungen und Vorerkrankungen"** von Garber S, Brunner JO, Heller AR et al. (2023) in *Die Anaesthesiologie.*
Beitrag und Zusatzmaterial stehen Ihnen auf www.springermedizin.de zur Verfügung. Bitte geben Sie dort den Beitragstitel in die Suche ein.

Tabelle S1: Mortalität (Mittelwert und Standardabweichung) je Patientengruppe und Steuerungspolitik in den betrachteten Zeitpunkten (Realsimulation)

	t	Politik 0	Politik 1	Politik 2	Politik 3	Politik 4	Politik 5
Alle Patienten	0	0,35 (0,06)	0,35 (0,06)	0,28 (0,06)	0,28 (0,06)	0,34 (0,06)	0,34 (0,06)
Patienten ohne Beeinträchtigung/Vorerkrankung	0	0,16 (0,08)	0,16 (0,08)	0,16 (0,08)	0,16 (0,08)	0,16 (0,08)	0,16 (0,08)
Patienten mit Beeinträchtigung/Vorerkrankung	0	0,44 (0,08)	0,44 (0,08)	0,35 (0,08)	0,35 (0,08)	0,43 (0,08)	0,43 (0,08)
Alle Patienten	1	0,35 (0,06)	0,29 (0,06)	0,29 (0,06)	0,25 (0,06)	0,34 (0,06)	0,28 (0,06)
Patienten ohne Beeinträchtigung/Vorerkrankung	1	0,16 (0,09)	0,16 (0,08)	0,16 (0,08)	0,16 (0,08)	0,16 (0,08)	0,16 (0,08)
Patienten mit Beeinträchtigung/Vorerkrankung	1	0,44 (0,08)	0,36 (0,08)	0,36 (0,08)	0,32 (0,08)	0,43 (0,06)	0,35 (0,06)
Alle Patienten	2	0,35 (0,06)	0,26 (0,06)	0,30 (0,06)	0,23 (0,05)	0,34 (0,06)	0,25 (0,06)
Patienten ohne Beeinträchtigung/Vorerkrankung	2	0,16 (0,09)	0,16 (0,07)	0,16 (0,08)	0,16 (0,07)	0,16 (0,08)	0,16 (0,07)
Patienten mit Beeinträchtigung/Vorerkrankung	2	0,44 (0,08)	0,32 (0,08)	0,37 (0,08)	0,29 (0,08)	0,43 (0,08)	0,31 (0,08)
Alle Patienten	3	0,35 (0,06)	0,23 (0,05)	0,30 (0,06)	0,22 (0,05)	0,34 (0,06)	0,23 (0,05)
Patienten ohne Beeinträchtigung/Vorerkrankung	3	0,16 (0,09)	0,16 (0,07)	0,16 (0,08)	0,15 (0,07)	0,16 (0,08)	0,15 (0,07)
Patienten mit Beeinträchtigung/Vorerkrankung	3	0,44 (0,08)	0,30 (0,08)	0,37 (0,08)	0,28 (0,08)	0,43 (0,08)	0,29 (0,08)

Tabelle S2: Durchschnittliche Anzahl an Patienten auf der Intensivstation je Patientengruppe und Steuerungspolitik in den betrachteten Zeitpunkten (Realsimulation)

	t	Politik 0	Politik 1	Politik 2	Politik 3	Politik 4	Politik 5
Patienten ohne Beeinträchtigung/Vorerkrankung	0	18,0	18,0	21,0	21,0	19,7	19,7
Patienten mit Beeinträchtigung/Vorerkrankung	0	42,0	42,0	39,0	39,0	40,3	40,3
Patienten ohne Beeinträchtigung/Vorerkrankung	1	18,0	20,9	20,7	23,9	19,6	22,6
Patienten mit Beeinträchtigung/Vorerkrankung	1	42,0	39,1	39,3	36,1	40,5	37,4
Patienten ohne Beeinträchtigung/Vorerkrankung	2	18,0	23,8	20,4	26,6	19,4	25,6
Patienten mit Beeinträchtigung/Vorerkrankung	2	42,0	36,2	39,6	33,4	40,6	34,5
Patienten ohne Beeinträchtigung/Vorerkrankung	3	18,0	26,6	20,2	29,0	19,2	28,3
Patienten mit Beeinträchtigung/Vorerkrankung	3	42,0	33,4	39,8	31,0	40,8	31,7
